# Integrating metabolomics and transcriptomics to analyze differences in muscle mass and flavor formation in *Gayal* and yellow cattle

**DOI:** 10.3389/fvets.2025.1581767

**Published:** 2025-05-14

**Authors:** Lin Han, Runqi Fu, Binlong Fu, Qian Li, Ye Yu, Huan Gao, Jiawei Zhang, Min Qi, Chunjia Jin, Shengyong Mao, Jing Leng

**Affiliations:** ^1^Faculty of Animal Science and Technology, Yunnan Agricultural University, Kunming, China; ^2^Key Laboratory of Animal Nutrition and Feed Science of Yunnan Province, Yunnan Agricultural University, Kunming, China; ^3^Centre for Ruminant Nutrition and Feed Engineering Technology Research, College of Animal Science and Technology, Nanjing Agricultural University, Nanjing, China

**Keywords:** *Gayal*, meat quality, regulation mechanism, transcriptome, metabolome

## Abstract

Beef flavor is affected by muscle metabolites and their related regulatory genes, and the molecular regulatory mechanisms vary among different beef breeds. To provide some new ways to improve meat quality and cattle breed improvement, 24-month-old *Gayal* (*n* = 8) and yellow cattle (*n* = 8) were selected for comparison in this study. The result revealed that the longissimus dorsi muscle fiber diameter, protein content and a-value of *Gayal* were significantly higher than that of yellow cattle, but the fat content was lower than that of yellow cattle. Furthermore, *Gayal* meat contained notably higher levels of polyunsaturated fatty acids (PUFA) and n-3PUFA than that of yellow cattle, and also had better levels of flavor amino acids (FAAs) and sweet amino acids (SAAs), which contribute to the flavor of beef. Through comprehensive analysis of transcriptomics and metabolomics, we detected a total of 109 markedly different metabolites (DEMs) and 1,677 differentially expressed genes (DEGs) in the pectoral muscles of the two breeds. Further analysis indicated that amino acid and lipid metabolism might be the key factors contributing to the differences in meat quality and flavor between *Gayal* and yellow cattle, involving metabolites such as L-2-aminobutyric acid, L-glutamic acid, L-glutamine, L-serine, betaine, pantothenic acid, and taurine. Through correlation analysis, we identified genes highly associated with flavor amino acids (*GSTM3*, *GSTT2*), muscle development (*FGF10*, *EIF4EBP1*, *PPP2R2C*), and lipid metabolism (*CYP4A22*, *ACOX3*, *PLIN1*, *ADH6*, *CNDP1*, *LPAR*3, *BRCA1*, *ADIPOQ*, *FABP3*) related essential regulatory genes and constructed a gene-metabolite interaction network for meat quality and flavor formation in *Gayal*. In summary, it was shown that significant differences in muscle metabolites between *Gayal* and yellow cattle, especially in amino acid and lipid metabolism, may be the major reason for the differences in quality and flavor between the two types of beef. This study provides a theoretical basis for further exploring the molecular regulatory mechanisms of the differences in beef quality and flavor between *Gayal* and yellow cattle, and provides a reference for the development and genetic breeding of high-quality cattle breeds.

## 1 Introduction

Livestock meat is a major source of protein and a vital component of the human diet, directly influencing overall health. Beef in particular is of significant nutritional value, containing not only high-quality proteins such as essential amino acids, but also rich vitamins, minerals and other important nutrients (1). Considering the flavor of beef and health factors, a remarkable increase in beef consumption has been observed. According to the Food and Agriculture Organization (FAO), global meat production has more than tripled over the past 50 years, with beef and buffalo meat production more than doubling since 1961 (2). In China, beef consumption has risen by 58% from 2000 to 2021 (3). The USDA projects that China’s beef market revenue will grow from USD 88.88 billion in 2023 to USD 124.29 billion by 2030, reflecting a compound annual growth rate of 4.9% (4). To meet the increasing demand, the cattle industry resorted to nutritional modification or introduction of cattle breeds with fast growth rate and high meat yield. However, these nutritionally regulated or imported cattle breeds are less resistant to disease and are susceptible to changes in the environment and feeding conditions. These factors may adversely affect their growth, development, and meat quality (5). As a result, they could potentially influence consumers’ choice of beef products. With the improvement of living standards, consumer demand for beef has shifted from quantity-based to quality-based, with more emphasis on quality, flavor and safety (6). However, how to improve meat quality while maintaining growth rate remains a major challenge for the domestic cattle industry. Research has shown that genetic selection is one of the effective means of improving meat quality (7). Therefore, an in-depth understanding of the genetic background and molecular markers for beef quality traits is crucial for assisted breeding of high-quality cattle.

Meat quality is a complex trait that is affected by a variety of physicochemical characteristics such as pH, tenderness, meat color, number of muscle fibers, intramuscular fat (IMF) content, fatty acid composition, and sensory quality (8). Transcriptome sequencing is one of the major methods to correlate biological phenotypes with molecular mechanisms. For instance, RNA-seq technology was used to screen fatty acid transporter candidate genes such as CD36, *SLC27A1*, and *FABP3*, whose differential expression may affect fatty acid composition (9). Research indicated that differentially expressed genes (DEGs) in bovine longissimus dorsi (LD) muscle and skeletal muscle regulate fatty acid composition of beef through fatty acid degradation, peroxisome proliferator-activated receptor (PPAR) and AMP-activated protein kinase (AMPK) signaling pathways (10). In addition, studies in metabolomics have found that metabolites such as acylcarnitines, free amino acids and glucuronic acid contribute to the color and oxidative stability of beef (11). Metabolomics is used as an effective research method to reveal the effects of gene regulation on changes in muscle metabolites. In recent years, non-targeted metabolomics has been widely applied to the study of beef metabolic mechanisms, and many studies have investigated the effects of nutrition, feeding management and breed on beef quality and metabolic profiles with remarkable results (5, 12, 13). In summary, transcriptomics and untargeted metabolomics techniques are effective and methodologically mature in beef research, and are able to effectively reflect the differences in metabolic profiles of bovine muscle. In addition, the joint analysis of the two can help to reveal the regulatory mechanisms of beef quality and flavor formation, and deepen the understanding of the regulatory mechanisms of meat quality (12–14).

China is rich in cattle breeds with the most cattle breeds in the world, including 53 local breeds, 7 domestic breeds and 13 imported breeds (15). As a distinctive grazing breed for high altitude areas of southwestern China, the *Gayal* (*Bos frontalis*) provides affordable and nutritious meat to local tribes and commands a high market price due to high demand for its traditional products (16, 17). Due to the great demand for traditional *Gayal* meat items, *Gayal* meat is pricey in the market (18). Besides, *Gayal* grow in a unique environment, which makes them exhibit strong immunity and unique growth mechanisms (19). Previous studies showed that the growth rate and muscle physicochemical properties of *Gayal* are superior to other cattle breeds (18, 20). More interestingly, the genes *KMT2C* and *SLC2A4RG* regulate cell proliferation, muscle development and energy metabolism through a gene regulatory network, further validating the advantages of *Gayal* in growth performance and meat quality (21). Mukherjee et al. (17) used Illumina-HiSeq technology for RNA-seq sequencing of LD muscle and identified 24 candidate genes associated with muscle development. These studies demonstrated that *Gayal* do possess unique growth and muscle development mechanisms, which are suitable as models for related studies. However, studies on muscle development and meat quality of *Gayal* are still relatively limited, and the molecular regulatory mechanisms are still unclear. Therefore, more research is needed to support this. In this study, *Gayal* and yellow cattle were selected to investigate the transcriptomic and metabolomic differences in meat quality traits such as muscle tenderness, muscle fiber density, IMF deposition and fatty acid content. In addition, we screened key regulatory pathways. It is anticipated that this research will play an important role in improving the quality of beef cattle and increasing the production of high-grade beef.

## 2 Materials and methods

### 2.1 Ethics statement headings

Animal experimental procedures were carried out in accordance with the Guide for the Care and Use of Laboratory Animals (72). All experiments involved in this study have been approved by the Institutional Committee for the Protection and Use of Animals of Yunnan Agricultural University under the approval number YNAU20220638-1.

### 2.2 Laboratory animals and feeding management

**Figure 1:**
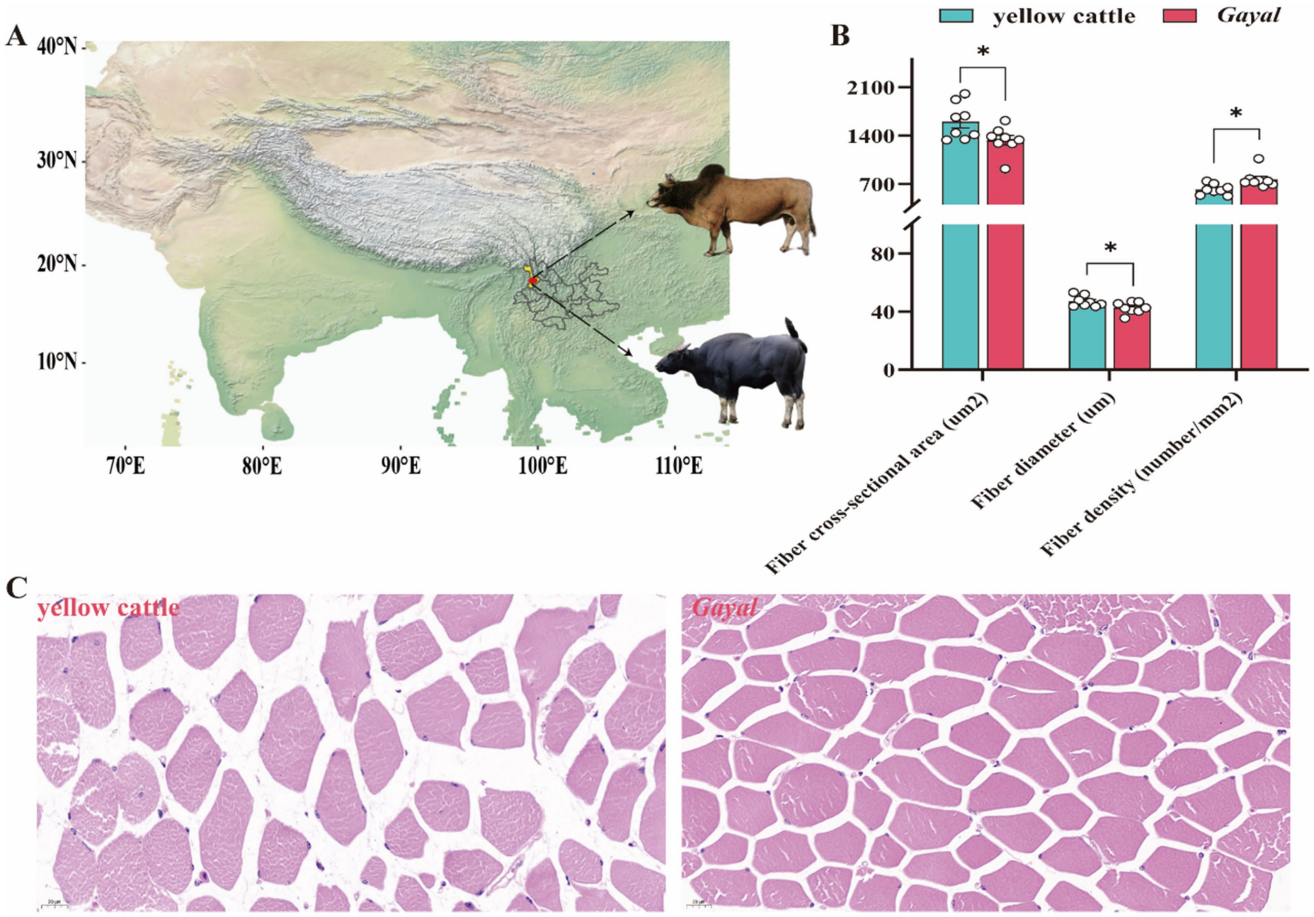
Comparison of distribution and muscle fiber index of *Gayal* and yellow cattle. **(A)** Map showing cattle in the geographic area where the test cattle grazed. **(B)** Comparison of myofiber morphometric characteristics between *Gayal* and yellow cattle. * indicate significant differences (*p* < 0.05) between *Gayal* and yellow cattle. **(C)** Tissue sections of the latissimus dorsi muscle of *Gayal* and yellow cattle were stained with hematoxylin and eosin (HE).

This study was conducted at the experimental pasture of the Phoenix Mountain *Gayal* Breeding and Expansion Base in Lushui City, Nujiang Prefecture, Yunnan Province, China. The pasture is located at an altitude of 2,700 meters (Figure 1A). A total of 16 bulls were selected for this study, including 8 male healthy *Gayal* (*Bos frontalis*) and 8 male healthy local yellow cattle (*Bos taurus*) from the Bilu Xueshan herd. Cattle were divided into two groups based on their similar age and weight: the *Gayal* group and the local yellow cattle group. The selection process lasted for over a month due to the semi-wild nature of these animals, making the procedure more challenging. During the experimental period, all cattle were allowed to graze in the trail pasture throughout the day and had unrestricted access to water and salt blocks. The cattle completed the entire 45-day experimental period in good health, without exhibiting any major symptoms that could have affected the results.

### 2.3 Sample collection

At the end of the experiment, all cattle were slaughtered, and the longissimus dorsi (LD) muscle samples (ribs 12–13) were collected and divided into two parts: one part was stored in liquid nitrogen for transcriptome and metabolome assays, and the other part was stored in dry ice for chemical analyses. The muscle samples were then stored at 4°C for 24 h for meat quality assessment. Moreover, we collected samples of the longest muscles of the back measuring 3 × 3 cm and treated them with fixation in 4% paraformaldehyde solution.

### 2.4 Measurement of LD muscle meat quality

The pH of the LD muscle was measured 45 min and 24 h postmortem using a portable pH meter (PHBJ-260, INESA Scientific Instrument, Shanghai, China). Flesh color parameters evaluated using a colorimeter (CR-410, Konica Minolta, Tokyo, Japan) - luminance (L*), redness (a*) and yellowness (b*) - were measured at three different sample locations. Shear force and drip loss were assessed following the method of Wang, An et al. (12). Shear force was measured as described in Shackelford et al. (22). In brief, meat samples with fat and connective tissue removed were heated in a water bath at 80°C to an internal temperature of approximately 71°C before being removed and cooled at 4°C. Samples with a diameter of 1.27 cm were then taken for shear force measurements along the longitudinal direction of the muscle fibers, using the C-LM3B device. Each sample was measured 6 times and finally the average value was taken. Drip loss was measured as follows: first, the muscle was trimmed along the direction of the muscle fibers to make strips of meat measuring 2 cm × 2 cm × 4 cm, and the initial weight (W1) was recorded (15). Subsequently, one end of the meat strip was suspended by a hook in a numbered plastic bottle and stored in a refrigerator at 4°C for 24 h. The weight of the meat strips (W2) was subsequently weighed again, and then the drip loss was calculated according to the following formula: drip loss (%) = [(W1 - W2) / W1] × 100, and the measurements were repeated four times for each sample, and the results were averaged. Samples (approximately100 g) were vacuum-sealed in polyethylene bags and cooked in a water bath at 80°C. Once the internal temperature reached 70°C, samples were cooled to room temperature. Cooking loss was calculated as: ((W1 − W2)/W1) × 100%, where W1 and W2 represent sample weights before and after cooking (23). Moisture, crude ash, IMF, and protein content were determined using AOAC (24) methods. Amino acids (AAs) content in freeze-dried BF muscle (1.5 g) was analyzed following GB 5009. 124-2016, with modifications. Samples were hydrolyzed in 6 mol/L HCl at 110°C for 22 h under nitrogen, centrifuged, and dried with an evaporator. Residues were dissolved in sodium citrate buffer and filtered through 0.22-μm membranes before analysis using an automatic amino acid analyzer (Sykam S-433D, Sykam Scientific Instruments, Beijing, China). Fat acids (FAs) content in LD muscle was analyzed using LC–MS/MS, following a modified protocol (15). Approximately 80 mg of muscle tissue was homogenized in liquid nitrogen, mixed with water, and vortexed. Besides, the fixed muscle tissue samples were sent to Wuhan ServiceBio Technology Co. for paraffin embedding and sectioning, and stained with hematoxylin–eosin (H&E) staining method. Subsequently, the morphological characteristics of muscle fibers and their areas were quantitatively analyzed using CaseViewer software (2.4.0.119028).

### 2.5 Metabolites extraction and data analysis

The LC–MS raw data were imported into the metabolomics software Progenesis QI (Waters Corporation, Milford, USA) for baseline filtering, peak identification, integration, retention time correction, and peak alignment, and finally a data matrix containing retention time, mass-to-charge ratio, and peak intensity was generated. Metabolites were identified by matching with HMDB,^1^ Metlin^2^ and majorbio’s own database. The searched data matrix was uploaded to the majorbio cloud^3^ for analysis. The data matrix was first pre-processed as follows: the 80% rule was used to remove missing values, i.e., variables with more than 80% of non-zero values in at least one set of samples were retained in the data matrix, and then the missing values were filled in (the smallest values in the original matrix were filled in the missing values). In order to minimize the errors brought by the sample preparation and instrumental instability, the response intensities of the sample peaks of the mass spectrometry were normalized by the sum-normalization method, and the normalized data matrix was obtained. The normalized data matrix was obtained. At the same time, variables with relative standard deviation (RSD) > 30% were deleted and log10 logarithmised to obtain the final data matrix for subsequent analyses. Then, the data matrices were uploaded to the majorbio cloud platform (25) for further analysis, and principal component analysis (PCA) and orthogonal least partial squares-discriminant analysis (OPLS-DA) were performed using the ropls package (Version 1.6.2) for R. The stability of the model was assessed by seven cycles of interactive validation. Significantly differentially expressed metabolites (DEMs) were screened based on the variable weight values (VIP) of the OPLS-DA model and Student’s t-test *p*-values (VIP > 1, *p* < 0.05, |Fold change| ≥ 1). The DEMs between the two groups were mapped to their respective biochemical pathways using metabolic enrichment and pathway analysis, based on the KEGG database.^4^ These metabolites were classified according to the pathways they are associated with or the functions they fulfill. Enrichment analysis was conducted to determine whether a particular functional node was represented within a group of metabolites. The approach extended the annotation of individual metabolites into the collective annotation of metabolite groups. Enrichment analysis was performed using the Python package “scipy.stats”,^5^ identifying the most relevant biological pathways corresponding to the experimental treatments.

### 2.6 Gene extraction and transcriptome analysis

Total RNA purification, reverse transcription, library construction and sequencing were performed at Shanghai Marjorie Biomedical Biotechnology Co. Ltd. (Shanghai, China) according to the manufacturer’s instructions. Total RNA from muscle was extracted using QIAzol Lysis Reagent (Qiagen, Germany). RNA quality was examined by a 5,300 Bioanalyzer (Agilent Technologies, USA) and a NanoDrop ND-1000 (Thermo Fisher Scientific, USA). Only high-quality RNA samples (OD260/280 = 1.8 to 2.2, OD260/230 ≥ 2.0; RQN ≥ 6.5) were used to construct sequencing libraries. Muscle RNA-seq transcriptome libraries were prepared using 1 μg of total RNA according to the Illumina® Stranded mRNA Prep, Ligation (San Diego, CA) method. Sequencing was performed on the NovaSeq X Plus platform (Illumina, Inc.) using the NovaSeq Reagent Kit. After quality control, clean reads were aligned to *Bos taurus* (GCF_002263795.3) in targeted mode using HISAT2 software (26). Subsequently, expression levels for each transcript were calculated based on TPM values per million mapped reads using StringTie and Ballgown software (26). Genes that were only expressed in at least 50% of the bovine samples in each group were included in subsequent analyses. For muscle transcriptome data, *p*-values were corrected by FDR (27). DESeq2 (28) considers differentially expressed genes (DEGs) with |log2FC| ≥ 1 and FDR < 0.05 to be significant. DEGs were subjected to principal component analysis (PCA), Gene Ontology (GO) functional annotation, and Kyoto Encyclopedia of Genes and Genomes (KEGG) pathway enrichment analysis using the Majorbio Cloud Platform (see Footnote 3) and R-based analytical tools. The results were comprehensively visualized.

### 2.7 Quantitative real-time PCR (RT-qPCR) for validation of RNA-Seq data

Total RNA was reverse transcribed using NovoScript®Plus All-in-one 1st Strand cDNA Synthesis SuperMix (gDNA Purge) (E047-01B; Novoprotein, Shanghai, China), and cDNA was synthesized according to the procedure provided by the manufacturer. For *GPX1*, *GSTA1*, *GSTM3*, *GSTT2*, *ADH6, COL1A2*, *COL1A1*, *FGF9*, *EIF4EBP1*, *FGF10*, *FABP3*, *ACOX3*, *GNGT2*, *TNXB*, *PIK3AP1*, *LPAR3*, *PLIN1*, *CYP4A22*, *ADIPOQ*, *THBS2*, *CREB5*, *PPP2R2C*, *GLULP*, *BRCA1*, and *β*-actin target genes, the cDNAs were synthesized using the Q-Tower3 (Analytik Jena AG, Jena, Germany) and NovoStart®SYBR qPCR SuperMix Plus (E096-01A; Novoprotein, Shanghai, China) for real-time quantitative PCR (qRT-PCR). Relative mRNA expression levels were analyzed by the 2^-ΔΔCT^ method and normalized using β-actin as an internal reference gene (13). Primers for differentially expressed genes were provided by Beijing Qingke Xinye Biotechnology Co. and are shown in Supplementary Table S1.

### 2.8 Statistical analysis

Meat quality data were analyzed using an unpaired two-tailed t-test in R (v4.3.1), and the results are shown as heatmaps. Correlations between DEGs and DEMs were assessed by Pearson correlation analysis. Using the psych package in R software (v4.3.1), the correlation and significance between differential genes and metabolites were calculated, and the data with |r| > 0.5 and *p* < 0.05 were screened out and imported into Cytoscape to draw network diagrams to explore the interaction between genes and metabolites. In addition, the correlation coefficients of genes, metabolites and meat quality characteristic indexes were calculated based on Pearson analysis using R software (v4.3.1), and all data were imported into Cytoscape for visualization.

## 3 Results

### 3.1 Determination of the LD muscle mass and muscle fiber index

**Figure 2:**
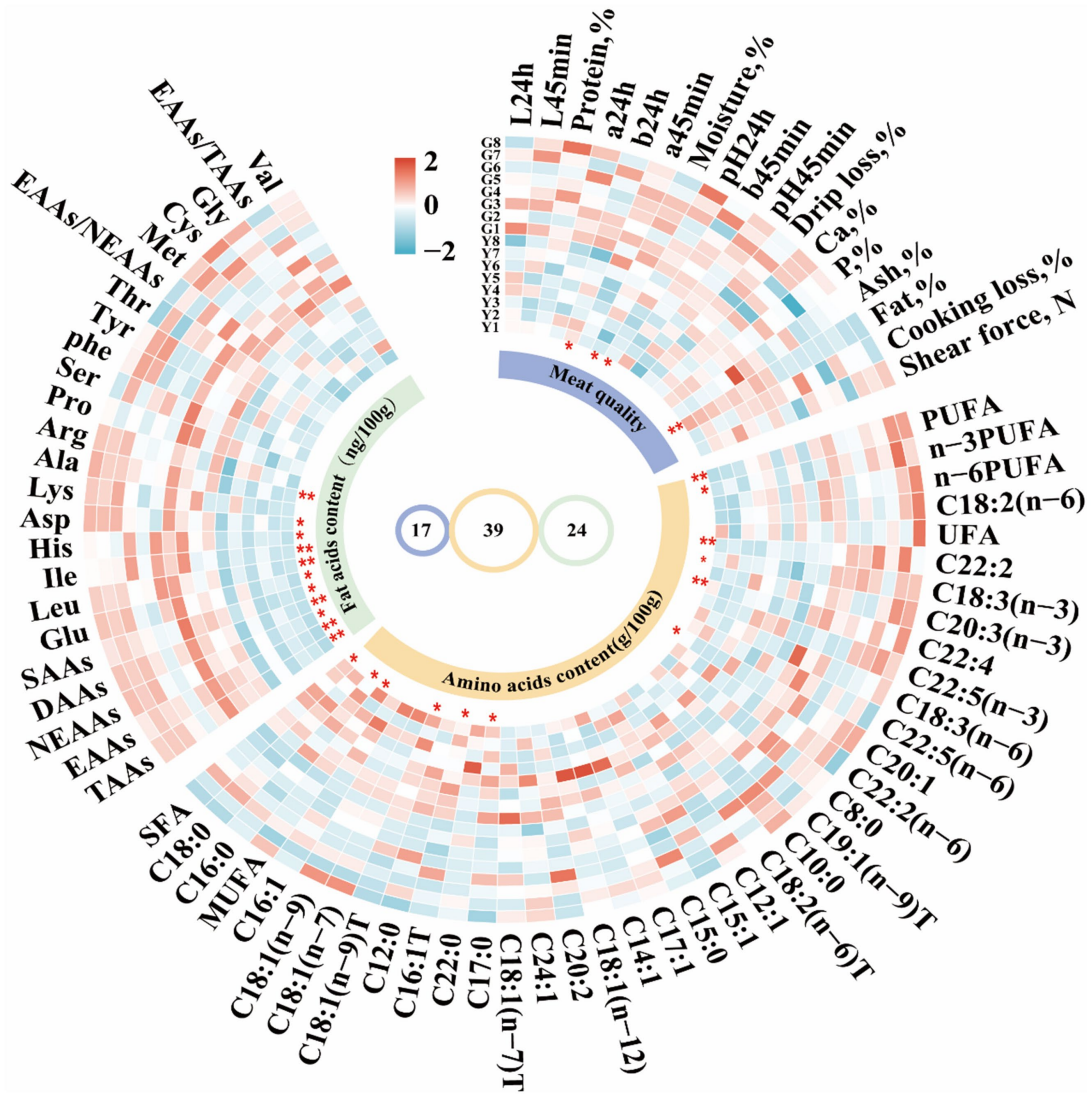
Cyclic heatmap analysis of meat characteristics and fatty acid and amino acid content of *Gayal* and yellow cattle using Complex Heatmap package for R 4.4.1. The fatty acid content of which saturated fatty acids (SFA) contained C8:0, C10:0, C12:0, C15:0, C16:0, C17:0, C18:0, and C22:0; monounsaturated fatty acids (MUFA) consisted of C12:1, C14:1, C15:1, C16:1, C16:1, C16:1 T, 17:1, C18:1(n-7)T, C18:1(n-12); C18:1(n-7)T, C18:(1n-9 T), C18:(1n-7), C18:(1n-9), and C24:1, whereas the unsaturated fatty acids (UFA) were derived by subtracting SFA from the total fatty acids. Polyunsaturated fatty acids (PUFA) included C18:2(n-6), C18:3(n-3), C18:3(n-6), C20:2, C20:3(n-3), C22:5(n-3), C22:5(n-6), and C22:2(n-6). T: trans. Amino acid analyses in the longissimus dorsi (LD) muscle showed that total amino acids (TAAs) consisted of essential amino acids (EAAs) such as His (Histidine), Ile (Isoleucine), Leu (Leucine), Lys (Lysine), Met (Methionine), Phe (Phenylalanine), Thr (Threonine), and Val (Valine), as well as non-essential amino acids (NEAAs) including Ala (Alanine), Asp (Aspartic acid), Arg (Arginine), Glu (Glutamate), Gly (Glycine), Ser (Serine), Tyr (Tyrosine), Pro (Proline), and Cys (Cysteine). DAAs (Delicious Amino Acids) consist of Asp., Glu, Gly, Ala, and Ile; SAAs (Sweet Amino Acids) consist of Gly, Ala, Ser, Pro, Lys and Thr. Statistical significance is indicated by * (*p* < 0.05) and ** (*p* < 0.01).

Comparative analysis of meat quality characteristics of *Gayal* and yellow cattle (Figure 1; Supplementary Table S2). Myofibers of *Gayal* and yellow cattle were stained using HE staining (Figure 1A) and it was observed under light microscope that nuclei stained blue and myofibers stained red. Comparing the myofibers of the two groups (Figure 1B), the diameter and cross-sectional area of the myofibers of *Gayal* were obviously lower than those of yellow cattle (*p* < 0.05), but the density of myofibers of *Gayal* was remarkably greater than that of yellow cattle (*p* < 0.05). Comparison of the LD muscle mass of the two groups of cattle (Figure 2) demonstrated that the protein content, a 45 min, and 24 h were considerably higher in *Gayal* than in yellow cattle (*p* < 0.05). *Gayal*, on the other hand, had considerably lower fat content and pH 45 min than that of the yellow cattle (*p* < 0.05, *p* < 0.01). In terms of L-value, b-value, shear force, cooking loss, drip loss, moisture, Ca, P and ash, no remarkable differences (*p* > 0.05) were shown between the two groups. To analyze the amino acid and fatty acid composition of the LD muscle, we conducted a quantitative metabolomics study on *Gayal* and yellow cattle. The results indicated that *Gayal* contained substantially lower levels of saturated fatty acids (SFA) and monounsaturated fatty acids (MUFA), and notably greater levels of polyunsaturated fatty acids (PUFA) and n-3PUFAs than yellow cattle (*p* < 0.05, *p* < 0.01). Of the 32 fatty acids identified, nine exhibited statistically significant variations between the two breeds (Figure 2; Supplementary Table S3). Specifically, *Gayal* exhibited considerably lower concentrations of C16:0, C17:0, C16:1 T, and C18:1(n-9)T, and considerably more C18:3(n-3), C22:3, and C22:4 (*p* < 0.05, *p* < 0.01) than yellow cattle. In terms of amino acid composition (Figure 2; Supplementary Table S4), the results of the study revealed that *Gayal* had dramatically greater levels of aspartic acid (Asp), glutamic acid (Glu), alanine (Ala), isoleucine (Ile), leucine (Leu), and histidine (His) than that of yellow cattle (*p* < 0.05, *p* < 0.01). This resulted in considerably higher (*p* < 0.05, *p* < 0.01) contents of essential amino acids (EAAs), non-essential amino acids (NEAAs), sweet amino acids (SAAs), delicious amino acids (DAAs), and total amino acids (TAAs) in the muscles of *Gayal* than that of the yellow cattle (*p* < 0.05, *p* < 0.01). Furthermore, The EAAs/TAAs and EAAs/NEAAs ratios of *Gayal* LD muscle, in this experiment, were 44.43 and 77.98%, respectively. These discrepancies in amino acid composition may contribute to the unique flavor of *Gayal* meat.

### 3.2 Transcriptome analyses of LD muscle

**Figure 3:**
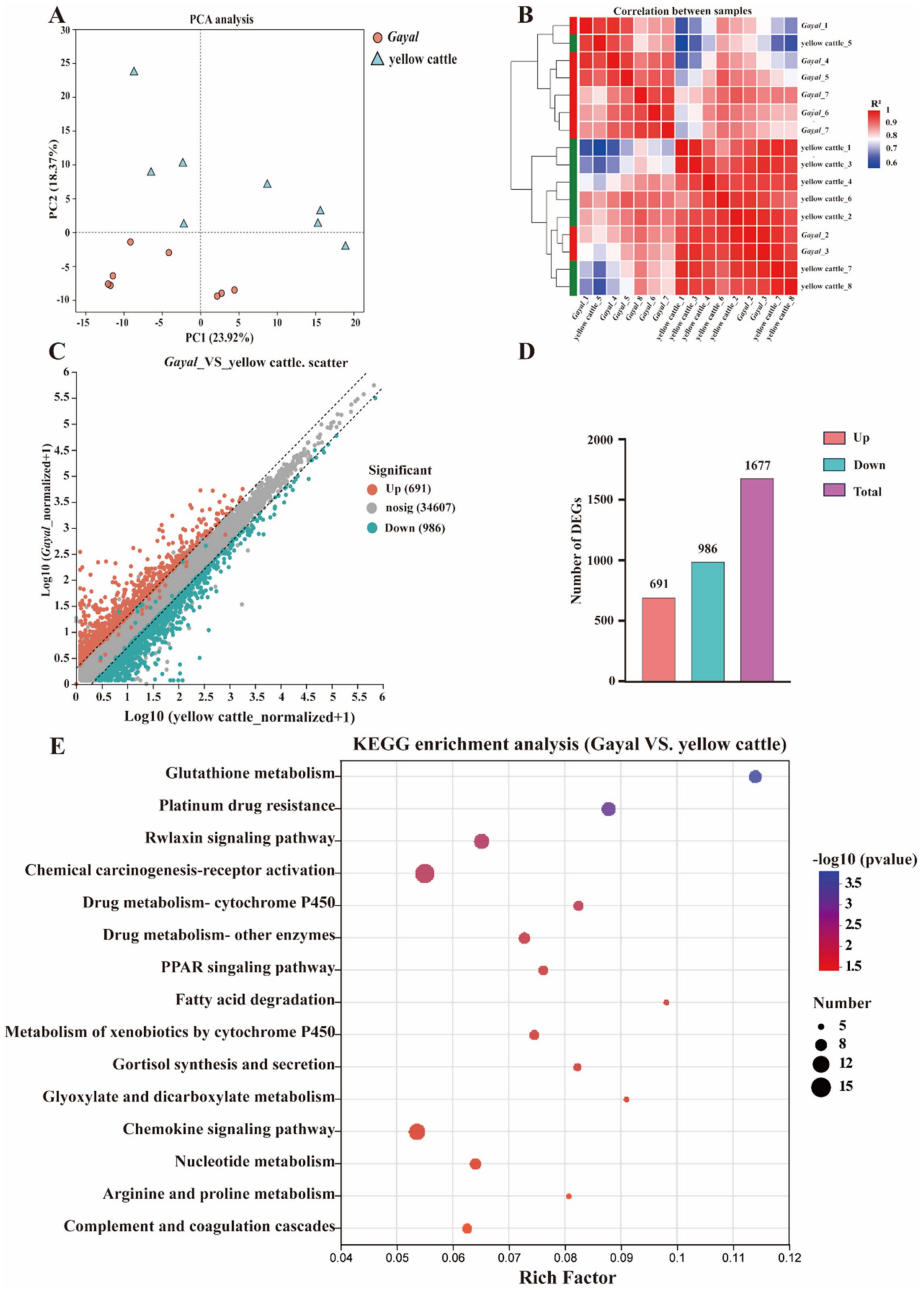
Transcriptomic profiling of longissimus dorsi (LD) muscle. **(A)** PCA visualizes transcriptomic differences. **(B)** Heatmaps of DEGs in colon and PM muscle. **(C)** Distribution of DEGs in the tissues. **(D)** The number of upward and downward DEGs. **(E)** KEGG analyses of DEGs indicate pathway enrichment.

**Figure 4:**
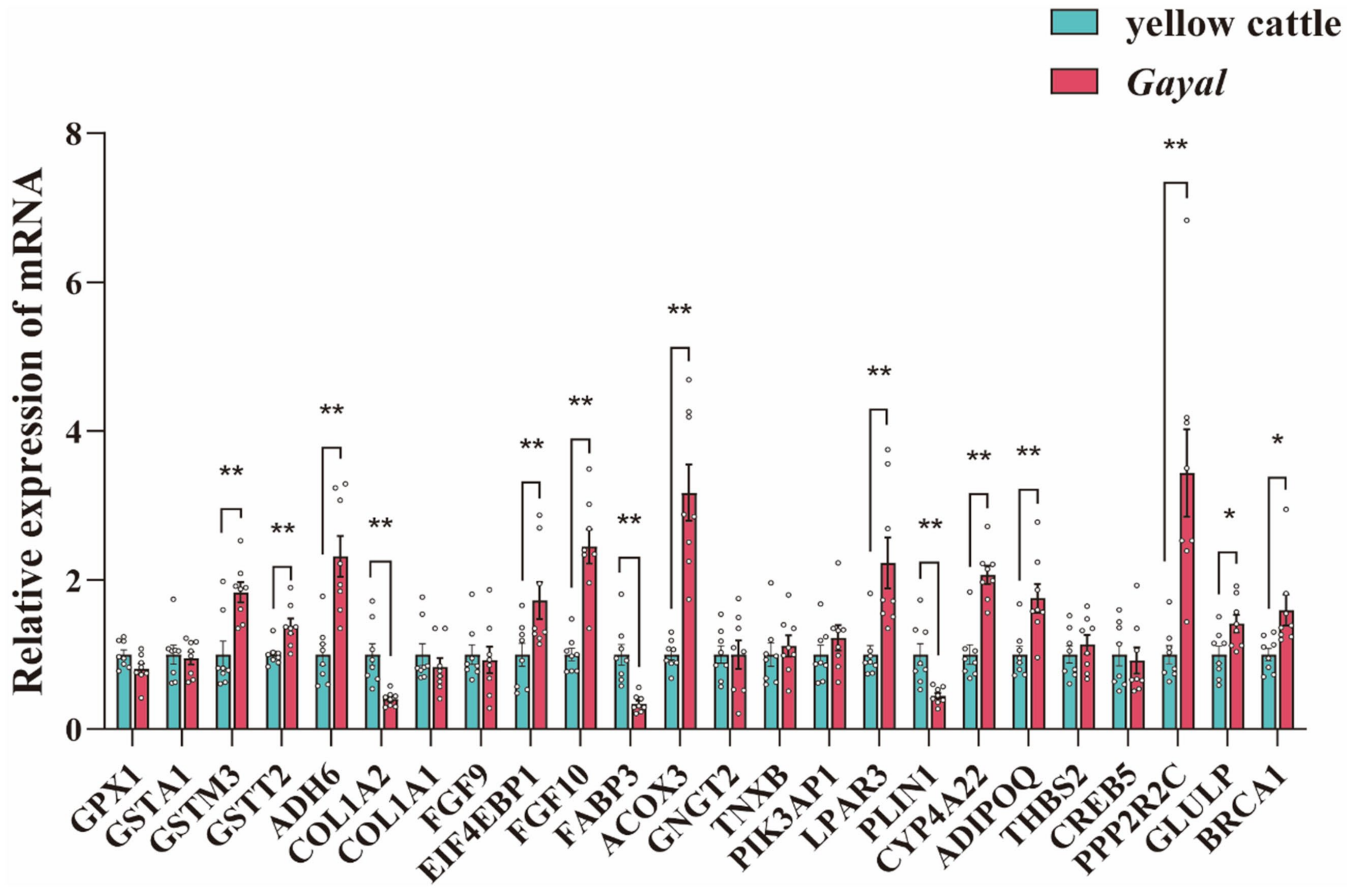
qRT-PCR was performed to validate DEGs in the muscles of longissimus dorsi (LD) of *Gayal* and yellow cattle. The results were analyzed by one-way ANOVA test in SPSS 20.0 (SPSS INC, USA). *GPX1*, Glutathione Peroxidase1; *GSTA1*, Glutathione S-Transferase Alpha 1; *GSTM3*, Glutathione S-Transferase Mu 3; *GSTT2*, Glutathione S-Transferase Theta 2; *ADH6*, Alcohol Dehydrogenase 6; *COL1A2*, Collagen Type I Alpha 2 Chain; *COL1A1*, Collagen Type I Alpha 1 Chain; *FGF9*, Fibroblast Growth Factor 9; *EIF4EBP1*, Eukaryotic Translation Initiation Factor 4E-Binding Protein 1; *FGF10*, Fibroblast Growth Factor 10; *FABP3*, Fatty Acid Binding Protein 3 (Heart); *ACOX3*, Acyl-Coenzyme A Oxidase 3; *GNGT2*, Guanine Nucleotide Binding Protein (G Protein), Beta Subunit 2; *TNXB*, Tenascin XB; *PIK3AP1*, Phosphoinositide-3-Kinase Adapter Protein 1; *LPAR3*, Lysophosphatidic Acid Receptor 3; *PLIN1*, Perilipin 1; *CYP4A22*, Cytochrome P450, Family 4, Subfamily A, Polypeptide 22; *ADIPOQ*, Adiponectin, C1Q, Collagen Domain Containing; *THBS2*, Thrombospondin 2; *CREB5*, cAMP Responsive Element-Binding Protein 5; *PPP2R2C*, Protein Phosphatase 2, Regulatory Subunit B, Gamma; *GLULP*, Glutamine Synthetase; *BRCA1*, Breast Cancer 1. Statistical significance is indicated as * (*p* < 0.05), and ** (*p* < 0.01).

In transcriptome sequencing, a total of 110.29 Gb of clean data was obtained. The clean data of each sample in both groups reached more than 6.25 Gb, and the percentage of Q30 bases was above 95.5%, indicating minimal sequencing errors (Supplementary Table S5). The mapping rate to the *Bos taurus* reference genome was 86.67–97.01%, indicating a reliable genome comparison and supporting reliable transcript quantification (TPM). The samples of each variety were clustered together in our analysis of principal component analysis (PCA), with samples of *Gayal* and yellow cattle separated by PCA1 (Figure 3A). A similar trend was previously observed in the heat map (Figure 3B). Gene expression was tissue-specific, and RNA-seq analysis of both species yielded a total of 25,321 genes, including 23,923 known genes and 1,398 new genes. There were 1,677 DEGs in the muscle of *Gayal* compared to yellow cattle, including 691 up-regulated genes and 986 down-regulated genes (Figures 3C,D; Supplementary Table S6). To determine the function of DEGs between *Gayal* and yellow cattle, we per-formed GO and KEGG functional enrichment analysis. The results revealed that there were more functional items for biological processes and relatively few genes for cellular components and molecular functions. In both groups, many genes were greatly enriched for the bioprocesses collagen fibril organization (GO: 0030199), monocarboxylic acid metabolic process (GO: 0032787) and response to endogenous stimulus (GO: 0009719; Supplementary Figure S1), potentially underlying *Gayal*’s superior muscle fiber density and amino acid metabolism. In addition, we obtained a total of 25 KEGG signaling pathways by KEGG enrichment analysis of DEGs (Supplementary Table S7). Among them, the obviously enriched signaling pathways included glutathione metabolism, arginine and proline metabolism, nicotinate and nicotinamide metabolism, fatty acid degradation, PPAR signaling pathway and PI3K-Akt signaling pathway (Figure 3E). The KEGG pathway emphasizes fatty acid degradation and PPAR signaling - which is key to the elevated levels of *Gayal* PUFA/n-3 PUFA - as well as glutathione metabolism (involving GSTM3, GSTT2), which may contribute to the antioxidant capacity and stability of the flavor precursors (Supplementary Table S7). CYP4A22 (fatty acid omega-hydroxylation) and ACOX3 (branched-chain fatty acid oxidation) genes are central to these pathways. The qRT-PCR validation test revealed that the qRT-PCR results were in agreement with the trend of the RNA-seq data (Figure 4).

### 3.3 Metabolome analysis of LD muscle

**Figure 5:**
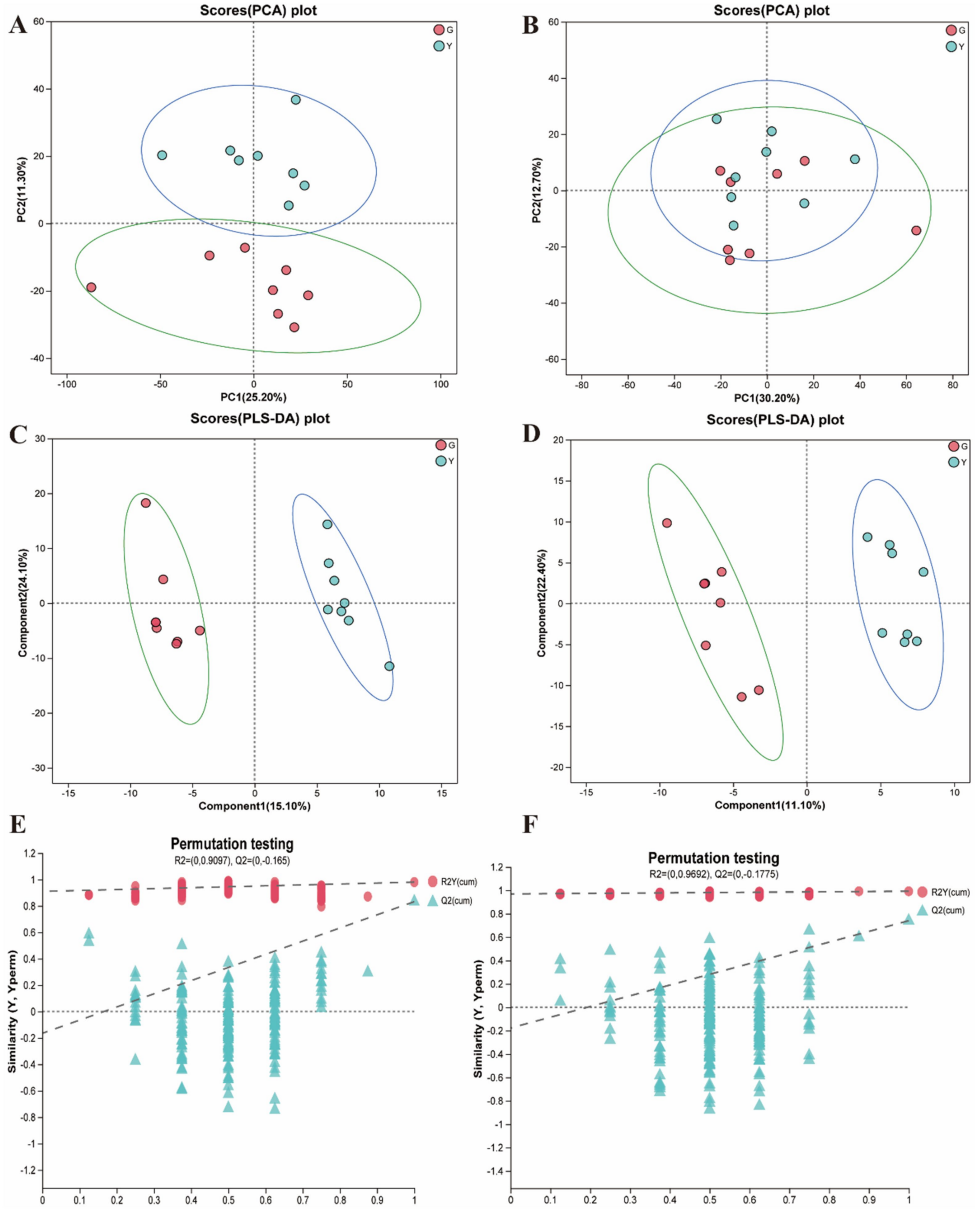
Quality control analysis of longissimus dorsi (LD) muscle metabolomics (*n* = 8). **(A)** PCA analysis in positive ion mode. **(B)** PCA analysis in negative ion mode. **(C)** OPLS-DA analysis in positive ion mode. **(D)** OPLS-DA analysis in negative ion mode. **(E)** The permutation test of OPLS-DA in positive ion mode. **(F)** The permutation test of OPLS-DA in negative ion mode.

**Figure 6:**
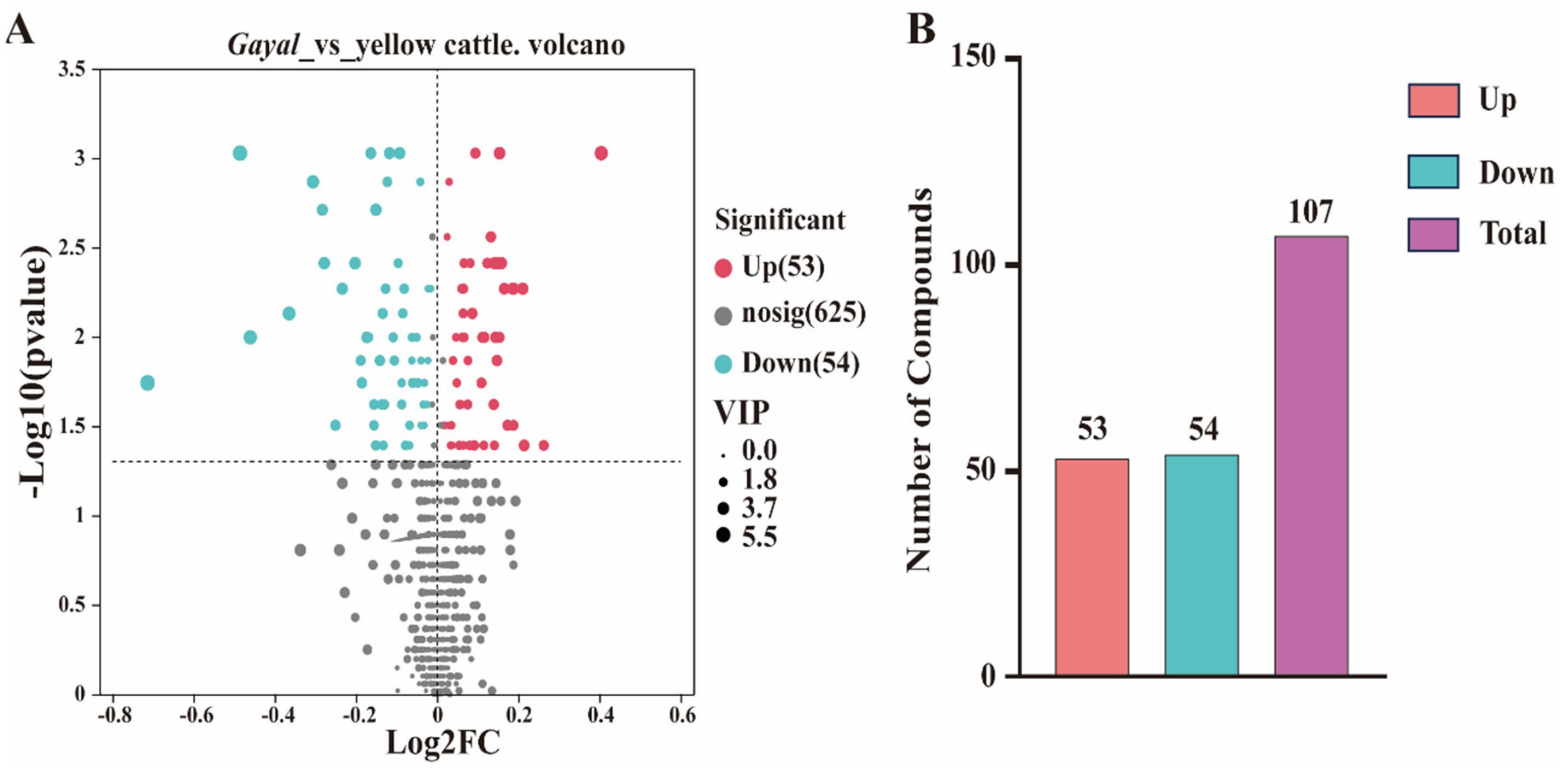
Number of metabolites differentially expressed in the LD muscle. **(A)** Volcano plot of differentially expressed genes. Gary is a non-significantly different gene, red and green are significantly different genes. **(B)** Histogram of differential gene expression statistics.

**Figure 7:**
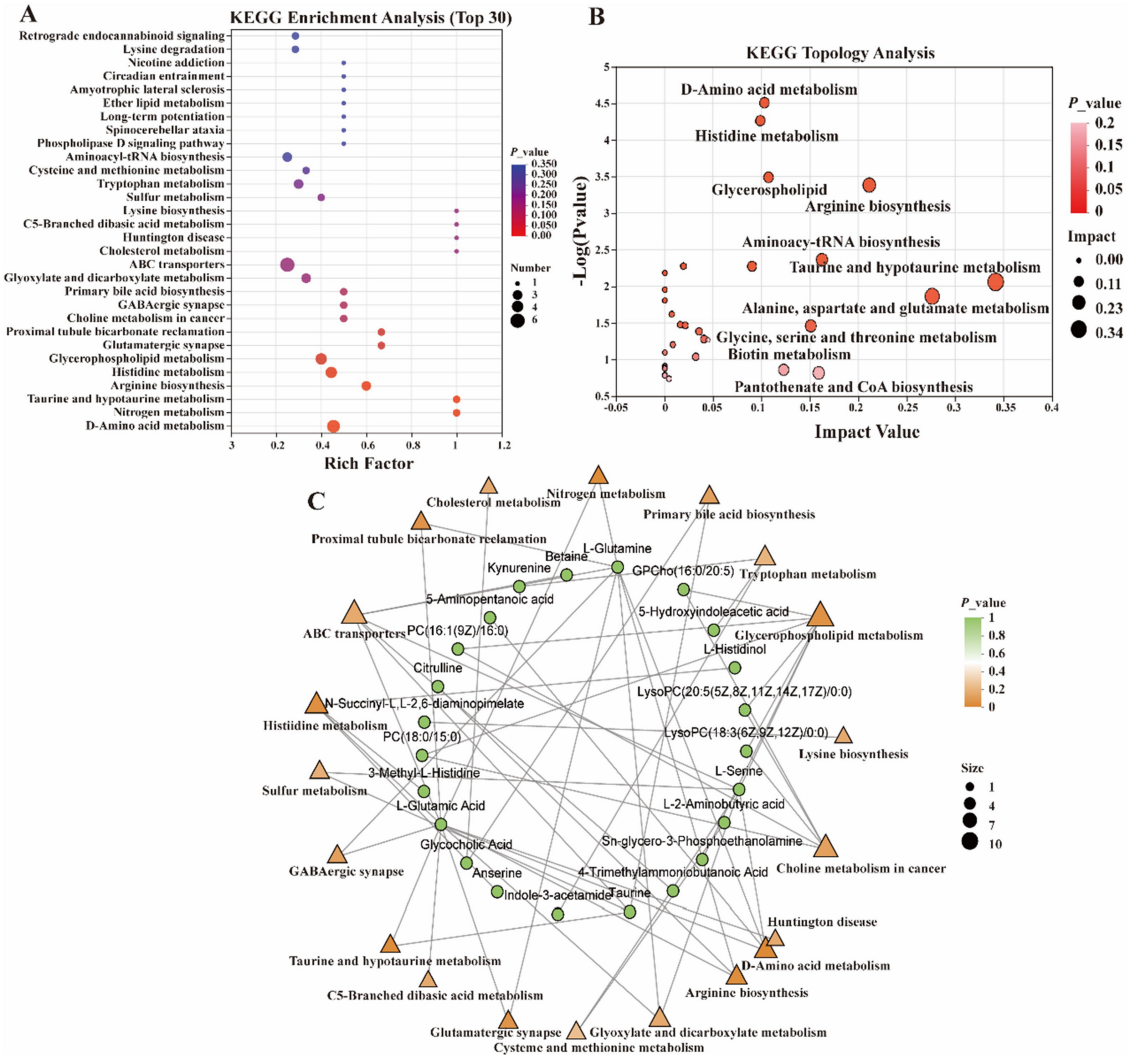
Functional analysis of differential metabolites. **(A)** Metabolite set enrichment analysis. **(B)** KEGG pathway analysis. **(C)** Interaction network of differential metabolites with significantly enriched terms.

In the present study, metabolites in two bovine LD muscles were analyzed by non-targeted liquid chromatography-mass spectrometry (LC–MS). PCA score plots showed significant differences in metabolite distributions between *Gayal* and yellow cattle muscle samples in positive and negative ion modes (Figures 5A,B). OPLS-DA modeling analysis further displayed that the metabolites in *Gayal* and yellow cattle muscle samples exhibited obvious separation under positive and negative ion modes (Figures 5C,D). In addition, the results of the permutation test illustrated that the OPLS-DA model did not display overfitting phenomenon (Figures 5E,F), and the R2 and Q2 values in the positive and negative ion modes were 0.9097 and −0.165, and 0.9692 and −0.1775, respectively, and the regression line displayed an increasing trend, which indicated that the model had a high degree of accuracy and stability. In conclusion, the results of PCA and OPLS-DA models of *Gayal* and yellow cattle muscle samples in positive and negative ion modes were stable and reliable, indicating that the samples have good reproducibility and can be used for subsequent analysis. In LC–MS analysis, a total of 732 metabolites were identified in muscle samples of *Gayal* and yellow cattle, and 107 DEMs were obtained according to the DEMs screening criteria. 53 DEMs were up-regulated and 54 DEMs were down-regulated in *Gayal* compared with those in yellow cattle (VIP ≥ 1, *p* < 0.05, Figures 6A,B; Supplementary Table S8). Among the DEMs, there were 34 lipid and lipid-like molecules, 9 organic acids and their derivatives, 6 organic oxides, 8 organic heterocyclic compounds, 1 nucleoside, nucleotide, and nucleotide, 12 organic nitrogen compounds, 2 organosulfur com-pound, 2 benzene compounds, 4 phenylketones and polyketones, 2 alkaloids and their derivatives, and 27 unclassified DEMs (Supplementary Table S8). Pairs of DEMs from the DEMs obtained from the LD muscles of *Gayal* versus yellow cattle demonstrated that 36 DEMs were up-regulated and 40 DEMs were down-regulated in the *Gayal* muscle in the positive ion mode, and 16 DEMs were up-regulated and 12 DEMs were down-regulated in the *Gayal* muscle in the negative ion mode (Supplementary Table S8). The contents of L-2-aminobutyric acid, L-glutamic acid, L-glutamine, N-Docosahexaenoyl Lysine, Do-cosapentaenoic acid (22n-3), L-serine, betaine, pantothenic acid, and taurine were significantly higher in *Gayal* muscle compared to that of yellow calves, whereas the contents of glycerophosphorylcholine and 4′- Aminoacetanilide were notably lower (Supplementary Table S8). Similarly, we enriched different metabolic pathways according to KEGG pathways, and nitrogen metabolism, arginine biosynthesis, histidine metabolism, taurine and taurine metabolism, pantothenic acid and coenzyme A biosynthesis, and glycerophospholipid metabolism were the important pathways between *Gayal* and yellow cattle (Figures 7A,B,C; Supplementary Table S9).

### 3.4 Correlation analysis

**Figure 8:**
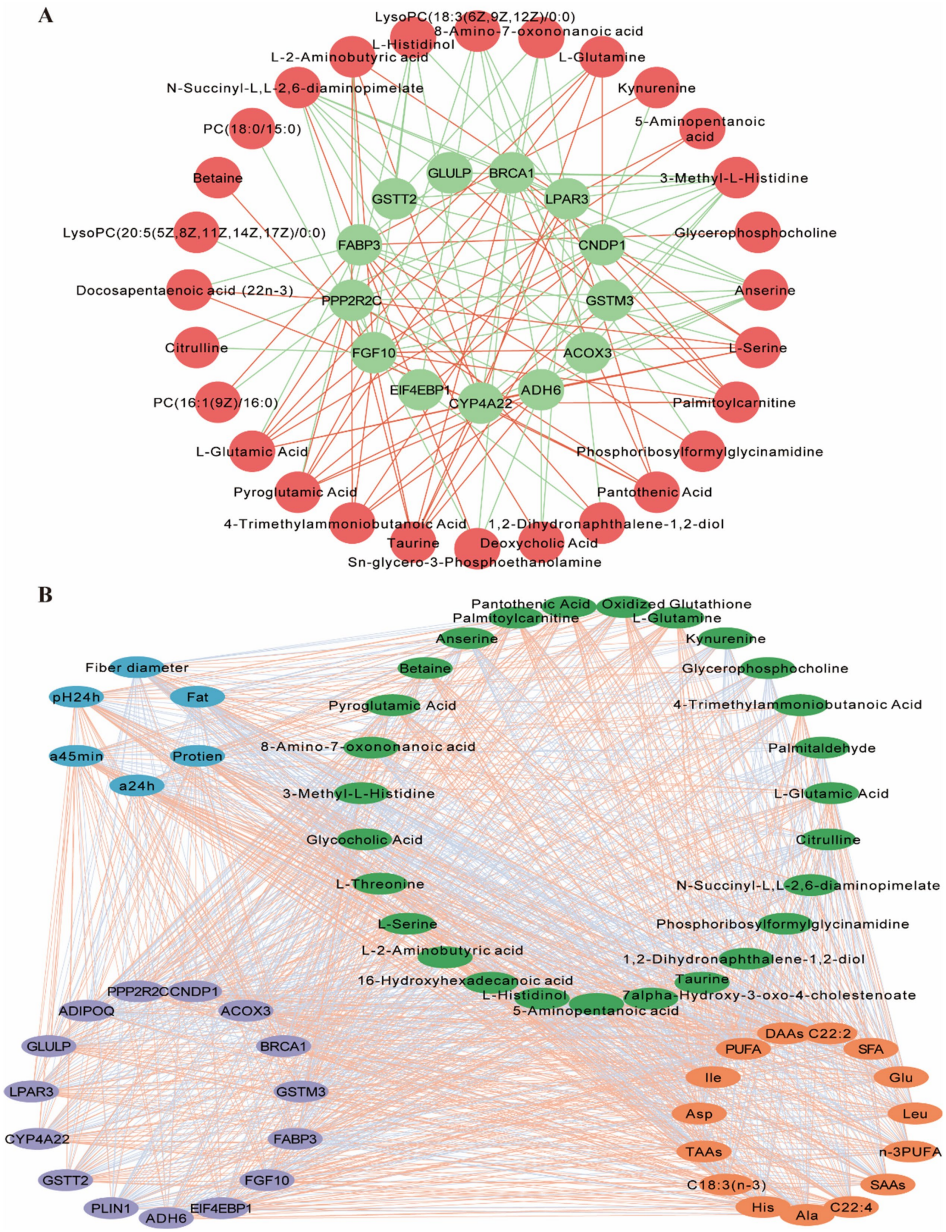
Correlation analysis between differentially metabolites (DEMs) and differentially expressed genes (DEGs) associated with meat quality and flavor. **(A)** Interaction network of meat-associated differential metabolites with DEGs. Red circles indicate differential metabolites and green circles indicate DEGs associated with meat quality differences. Red lines indicate positive correlations and blue lines indicate negative correlations. **(B)** Interaction networks of muscle differential physicochemical parameters, amino acids and fatty acids with differential genes and their metabolites. Correlations between them were calculated by R package psyche, and all data were imported into Cytoscape for plotting. Blue circles represent physicochemical parameters, green circles represent DEGs associated with meat quality, light brown circles represent differential genes associated with meat quality, and orange circles represent meat-differentiated amino and fatty acids. Red lines indicate positive correlations and green lines indicate negative correlations.

The integration of meat characteristics, metabolomics, and transcriptomics data from the LD muscle revealed significant correlations between meat color, protein content, DAAs, SAAs, PUFA levels, and genes and metabolites involved in amino acid and lipid metabolism pathways. Therefore, this study focused on analyzing amino acid and lipid-related pathways (Figure 8). We performed correlation analyses of metabolites and genes in these two types of pathways and screened for DEGs associated with DEMs of LD muscle mass and flavor. Pearson correlation analysis was used to assess the correlation between meat quality, DEMs and DEGs. Correlation coefficients greater than 0 indicated positive correlation and less than 0 indicated negative correlation. The correlations between meat quality and flavor-related amino acid and lipid DEMs and the corresponding DEGs are shown (Figure 8B). In addition, we analyzed specific metabolites and genes that might affect muscle quality and flavor, considered gene-metabolite pairs with correlation coefficients greater than 0.5 and *p* values less than 0.05 as strong correlation pairs, and mapped amino acid- and lipid-related gene-metabolite networks using Cytoscape to further explore their potential roles (Figure 8A). Based on the results of these analyses, we identified a network that may regulate *Gayal* meat quality and flavor formation (Figure 8), which majorly involves alanine, aspartate, and glutamate metabolism, histidine metabolism, nitrogen metabolism, glycerophospholipid metabolism, and other pathways associated with DEMs and DEGs. However, the regulatory network of beef quality and flavor formation is extremely complex, and the current study has only revealed some of the mechanisms, so further studies are needed to validate these findings and explore their mechanisms in depth.

## 4 Discussion

*Gayal* (*Bos frontalis*) is a distinctive semi-wild cattle breed distributed in high mountains and subtropical rainforests in the Nujiang River Basin of Yunnan Province in southwestern China, with bamboo as its staple food, which is considered a natural green food because it grows in a pollution-free environment (29). In recent years, comparison of different cattle breeds has become an effective method to study the merits of meat quality (30, 31). The present study indicated that there was no significant difference between *Gayal* and yellow cattle in terms of drip loss and cooking loss, suggesting that both have similar physical muscle characteristics. However, in terms of redness (a* value), the muscles of *Gayal* were considerably greater than those of yellow cattle, indicating that *Gayal* have more reddish meat color and are more marketable. This discrepancy may be attributed to the increased myoglobin content in *Gayal* in the hypoxic environment of the plateau. Particularly in anoxic environments, the enzymatic activity of *Gayal* muscle is enhanced, resulting in a reduced state of iron ions (Fe^2+^), which further deepens the meat color (32). These findings are consistent with experimentally observed results on muscle protein and AAs content and are similar to yak-related studies (32–34). Muscle pH decreased over time (35). However, due to glycogen depletion, the pH value almost ceased to decrease after 24 h. Low pH causes denaturation of myoplasmic and myofibrillar proteins in muscle fibers, which means reduced water holding capacity, color and palatability (36). We found that the pH 24 h value of *Gayal* was found to be considerably higher than that of yellow cattle, which contributes to the quality of *Gayal*. Myofiber characteristics are also an important factor in meat quality (30). Myofiber size is determined by the size of the muscle bundles, with larger myofibers leading to a thicker muscle cross-section (30). Also, the fineness of myofibers determines muscle tenderness: smaller myofibers usually mean lower shear and higher tenderness (30, 37, 38). Tenderness is a pivotal factor in meat consumer satisfaction and repeat purchase intention, which is directly correlated with consuming quality (30, 39, 40). The present study demonstrated that the myofiber diameter and cross-sectional area of LD muscles of *Gayal* were remarkably smaller than those of yellow cattle, whereas their myofiber density was remarkably larger than that of yellow cattle. Therefore, the shearing force of *Gayal* was slightly lower than that of yellow cattle, indicating that the LD muscle of *Gayal* was more tender and had superior eating quality than that of yellow cattle. Beef is an essential source of nutrients such as high-quality proteins and amino acids (13). Studies have shown that fatty acid and amino acid composition remarkably influenced the flavor, juiciness and nutritional value of meat (13, 30, 31). Nevertheless, comparative studies on *Gayal* and yellow cattle in terms of fatty acid and amino acid composition have not been reported. Among the fatty acids affecting beef flavor, unsaturated fatty acids (UFA) are in particular of importance, especially PUFA (41). Furthermore, considering that SFA probably increase total cholesterol and lower low-density lipoprotein cholesterol levels, leading to an increased risk of cardiovascular disease (CVD), numerous studies have recommended limiting their intake (42). In contrast, lower SFA and higher n-3 PUFA intake have been suggested to reduce the risk of CVD (43). In the present study, we found that the proportion of UFA was 53.42% in *Gayal* compared to 48.19% in yellow cattle, suggesting that the proportion of UFA is a key factor influencing intermuscular fat content. Furthermore, the n-3 PUFA in the diet is crucial for human health (44). Our experimental results also showed that *Gayal* contained substantially greater n-3 PUFA than yellow cattle, suggesting that *Gayal* meat may be more beneficial to human health. Additionally, alanine reacts with reducing sugars to form flavor compounds that elevate meat freshness (45). Catabolic metabolites (e.g., *α*-keto acids and aldehydes) of branched-chain amino acids (leucine and isoleucine) can further react with reducing sugars or fatty acids to generate compounds with distinctive flavor profiles (32, 46). Meanwhile, glutamine absorption and catabolism are essential for the formation and maintenance of myofibroblasts (47). In the present study, *Gayal* meat was observed to contain prominently higher levels of alanine, isoleucine, leucine, histidine and glutamate than that of yellow cattle, which resulted in increased levels of the DAAs and SAAs. This indicates that the nutritional value and flavor of *Gayal* meat meets consumer expectations. According to the quality standards of the Food and Agriculture Organization of the United Nations (FAO) and the World Health Organization (WHO), the recommended total EAAs is not less than 40% of the TAAs and 60% of the NEAAs. The EAAs/TAAs and EAAs/NEAAs ratios of *Gayal* meat, in this experiment, were 44.43 and 77.98%, respectively, which met the above recommended criteria, which further validated its potential as a source of high-quality protein. Moreover, the increased concentrations of EAAs, NEAAs and TAAs in LD muscle of *Gayal* contributed to the improvement of its nutritional value and flavor. However, future studies should delve into the potential mechanisms behind these changes to further reveal their role and value in meat quality improvement.

Meanwhile, LD muscles of *Gayal* and yellow cattle were collected for transcriptome and metabolome sequencing analysis to further assess the impact of their meat quality at the molecular level. Muscle development and lipid metabolism of livestock are core components of their growth and development, both of which are not only related to meat yield but also directly affect the economic value of the animal. Muscle growth is a complex multistage process regulated by a variety of factors, especially metabolic pathways and genes that are closely related to muscle development and lipid metabolism (30). In the present study, 1,677 DEGs were notably enriched in lipid metabolism-related pathways such as fatty acid degradation, PPAR, PI3K-Akt, glutathione metabolism, and arginine and proline metabolism. Activation of the fatty acid degradation pathway probably contributed to the improvement of fatty acid utilization efficiency in muscle and the reduction of fat deposition, thus enabling the semi-wild *Gayal* to adapt to the harsh environment through their own metabolic mechanisms. In particular, the PPAR pathway, as a ‘molecular switch’ of lipid metabolism, is crucial in the regulation of lipid metabolism, energy balance and insulin sensitivity (48). Furthermore, the high correlation between gene expression patterns and differences in fat deposition among breeds has been confirmed by several studies (13). It has been found that *GLULP* agonists (e.g., Exendin-4) were able to inhibit the expression of key factors (e.g., *MSTN*, atrogin-1, and *MuRF-1*) during muscle atrophy and activate the PKA and AKT pathways, thereby promoting muscle mass and function (30, 49). This finding suggests that by modulating these signaling pathways may be able to contribute to improved muscle development and meat quality. For example, the development of feed additives targeting *GLULP* agonists may improve muscle growth performance and meat quality in cattle. Additionally, antioxidant and cell metabolism-related genes such as *GSTM3* and *GSTT2* support normal physiological functions of muscle cells by regulating antioxidant responses, maintaining cellular metabolic homeostasis, and protecting cells from oxidative damage (50, 51). It has been shown that high expression of glutathione metabolism genes, such as *GSTM3* and *GSTT2*, to improve meat color and water retention may maintain muscle cell homeostasis by scavenging reactive oxygen species and delaying protein denaturation due to oxidative stress (52). Further analyses revealed that *PPP2R2C* affects muscle protein synthesis by dephosphorylating mTOR-related molecules (e.g., *4EBP1*), and may have an effect on muscle fiber type (e.g., fast versus slow muscle ratio), which in turn alters meat tenderness and shear force. Therefore, despite the lower IMF content of the *Gayal* in this study, their shear force was not significantly different from that of the yellow cattle. Researchers have found that fibroblast growth factor 10 (*FGF10*) regulates muscle cell proliferation and differentiation and promotes the formation of muscle tissue, which improves meat quality characteristics (53). Over-expression of *FGF10* reduces lipid accumulation in animal models (54). Lipid metabolism analysis revealed that the expression of relevant genes in fatty acid degradation and amino acid metabolism pathways was markedly correlated with meat quality, and that these genes may play a crucial role in lipid metabolism, meat quality, and flavor differences between Gaelic and yellow cattle. Our study further validated the importance of the *PPAR* pathway and its related genes in regulating differences in muscle lipid metabolism. *CYP4A22* belongs to the cytochrome P450 (CYP) family, which mainly catalyzes *ω*-hydroxylation of medium-chain fatty acids (e.g., lauric acid LA and myristic acid MA) involved in fatty acid metabolism and synthesis of bioactive substances (55). Studies showed that hydroxylases of the *CYP4A* family play an essential role in rat skeletal and arterial myocytes, producing 20-hydroxyeicosatetraenoic acid and eicosatrienoic acid, which further validates the potential function of *CYP4A22* in fatty acid metabolism (56). The *ACOX3*-encoded acyl-coenzyme A oxidase 3 is involved in the dehydrogenation of 2-methyl-branched-chain fatty acids in the peroxisome (57) and plays an important role in the regulation of chicken meat quality (58), oxidizing bovine straight-chain fatty acids (59), a result that has also been validated in beef cattle (13, 60). In addition to this, the genes *ADH6*, *EIF4EBP1*, *LPAR3* and *BRCA1* involved in UFA biosynthesis, amino acid metabolism and fatty acid degradation should be considered. We also found that these genes upregulated in *Gayal* were remarkably positively correlated with PUFAs, DAAs and SAAs and negatively correlated with adiposity. It indicated that these genes may promote muscle growth and development by reducing intramuscular lipid deposition, optimizing muscle fiber properties, and thus improving meat quality characteristics. However, at the same time, we found that most of the enriched genes in the PPAR signaling pathway were down-regulated in *Gayal*, and the star genes in this pathway (*PLIN1*, *ADIPOQ*, *FABP3*) have been extensively reported to be closely associated with IMF deposition (12, 13, 61). This result further validated the characterization of low-fat content in *Gayal* muscle. In summary, although the available evidence supports the potential benefits of these genes in improving flavor and health, further research and optimization of their taste is needed to meet the diverse needs of consumers.

Metabolomics has provided new perspectives for animal breeding and nutritional studies and has become one of the most active fields at present. In order to further reveal the phenotypic differences, we performed non-targeted metabolomic analysis on the LD muscle of two cattle breeds. In this study, the most abundant DEMs between the muscles of *Gayal* and yellow cattle were organic acids and their derivatives, most of which belonged to lipids, amino acids, peptides and analogs, and vitamins. In particular, changes in the concentrations of L-2-aminobutyric acid, L-glutamic acid, L-glutamine, and L-serine can be an essential indicator for assessing the nutritional value of meat (62, 63). Specifically, glutamine-supplemented diets improved meat color, mitigated the rapid decline in pH and reduced water loss, further improving meat quality (64). L-2-Aminobutyric acid, as a nitrogen-containing compound, promotes amino acid metabolism in muscle cells, regulates muscle structure and function, and consequently improves meat texture and tenderness (65). Glutamine is a precursor of glutamate and is involved in the maintenance of nitrogen balance and the promotion of protein synthesis (66). Studies also indicated that an adequate supply of serine is essential for muscle protein synthesis and renewal, maintaining good structure and elasticity of muscle fibers, which results in meat that is more tender when consumed. Conversely, meat tenderness is reduced remarkably when serine supply is insufficient (73). Additionally, glutamine contributes to fresh flavor (62). Betaine (also known as trimethylglycine) has demonstrated remarkable benefits for meat quality. Dong et al. (67) found that betaine supplementation improved growth performance, increased eye muscle area, reduced shear and drip losses, and improved muscle texture by increasing amino acid and fatty acid content in sheep. Additionally, betaine enhanced antioxidant activity in muscle, resulting in improved meat quality (68). These findings have also been validated in broiler studies (63). Meanwhile, lipids are important flavor precursors in raw meat and can be converted into volatile flavor compounds during meat processing. We found that N-docosahexaenoyl lysine and docosapentaenoic acid (22n-3) were significantly up-regulated in the muscles of *Gayal*, further validating the increased PUFA content in the muscles. Notably, Pantothenate (vitamin B5), a potential flavor precursor, was shown to be up-regulated in *Gayal* muscle. Pantothenate is involved in metabolic processes involving the metabolism of carbohydrates, fats and proteins and positively affects the flavor of meat (63). Taurine plays an important role in enhancing the nutritional value and flavor of meat with its antioxidant effects, stabilization of cell membranes, and involvement in lipid homeostasis (12). Studies showed that taurine supplementation improved the flavor of lamb (69). De et al. (70) reported that 0.5% taurine additive reduced the proportion of type IIb myofibrils in thigh muscles of broiler chickens and reduced glycolysis and attenuated protein denaturation, resulting in improved meat quality. In summary, this study revealed the potential of multiple metabolites in meat quality improvement, especially in the regulation of fatty acid and amino acid metabolism and meat flavor. Within the KEGG enrichment analysis, DEMs were predominantly enriched in pathways related to amino acid and lipid metabolism. Among them, nitrogen metabolism, taurine and subtaurine metabolism, ether lipid metabolism, arginine biosynthesis, histidine metabolism, D-amino acid metabolism and glycerophospholipid metabolism. These metabolic pathways may be involved in the regulation of *Gayal* LD muscle mass and flavor. Amino acid metabolism, taurine and taurine metabolism, and glycerophospholipid metabolism were found to be the key metabolic pathways involved in meat flavor formation (13, 63, 71). In addition, the literature showed that taurine and subtaurine metabolism, amino acid metabolism, and glycerophospholipid metabolism differed significantly in the muscles of different animal breeds and were important pathways affecting muscle flavor (13, 63). The results of our study are similar to those mentioned above. In summary, the DEMs and metabolic pathways obtained by metabolomics analysis were mainly involved in amino acid, lipid, and vitamin metabolism, suggesting that differences in amino acid, lipid, and vitamin metabolism may be the main cause of differences in dorsal muscle quality and flavor between *Gayal* and yellow cattle.

By comprehensive analysis, we discovered that the regulatory network of beef quality and flavor formation includes nitrogen metabolism; arginine biosynthesis; histidine metabolism; and glycerophospholipid metabolism, which includes the key DEMs and DEGs. however, we only understand a small part of it, and more research is needed to explore the finer regulatory network in the future.

## 5 Conclusion

In this study, the LD muscle mass of *Gayal* and yellow cattle was measured and analyzed comprehensively by combining transcriptomics and metabolomics. The results showed that *Gayal* muscle mass exhibited superior characteristics compared with that of yellow cattle, as evidenced by smaller muscle fiber diameters, redder meat color, and higher contents of protein, PUFA, DAAs, and SAAs. Through comprehensive analysis of transcriptomics and metabolomics, we identified 1,677 DEGs and 109 DEMs, and further screened for genes (e.g., *CYP4A22* and *ACOX3*) and metabolites (e.g., L-glutamate, L-glutamine, taurine, and betaine) related to *Gayal* meat quality regulation. There were significant correlations between these genes and metabolites, which may be jointly involved in the regulation of *Gayal* beef quality. Although *Gayal* beef is rich in a variety of essential amino acids and exhibits a unique flavor, its IMF content is relatively low due to its growth in a special environment and pasture-based feeding. However, the specific mechanism of how to effectively regulate the tenderness of *Gayal* beef, which is one of the key indicators of meat quality, has still not been fully explored. Therefore, the results of this study provide theoretical support for marker-assisted breeding and have important application prospects.

## Data Availability

The datasets presented in this study can be found in online repositories. The names of the repository/repositories and accession number(s) can be found at: https://www.ncbi.nlm.nih.gov/, PRJNA1216010; https://www.ebi.ac.uk/metabolights/, MTBLS12194.

## Footnotes

^1^ http://www.hmdb.ca/

^2^ https://metlin.scripps.edu/

^3^ cloud.majorbio.com

^4^ http://www.genome.jp/kegg/

^5^ https://docs.scipy.org/doc/scipy/
