# Supplementary material for: Integrating metabolomics and transcriptomics to analyze differences in muscle mass and flavor formation in Gayal and yellow cattle
Source: Front Vet Sci. 2025 May 14;12:1581767. doi: 10.3389/fvets.2025.1581767 (PMC12116499; doi:10.3389/fvets.2025.1581767)
Supplement: Supplementary file 1 [file Data_Sheet_1.DOCX]

Supplementary Material

# Supplementary Data

Supplementary material has been uploaded separately, including supplementary data sheets.

# Supplementary Figures and Tables

## Supplementary Tables

**Supplementary Table 1.** The information of primers for the genes in qPCR

| Gene Symbol | Forward primer | Reverse primer | Product length(bp) | Tm **(**℃**)** |
| --- | --- | --- | --- | --- |
| *β-actin* | GCGGCATTCACGAAACTACC | GCCAGGGCAGTGATCTCTTT | 148 | 60 |
| *CYP4A22* | CCCACTCCAATCATGGTGCT | CACAAAAGTCCTGGATCAGACA | 80 | 60 |
| *GPX1* | AACGTAGCATCGCTCTGAGG | GATGCCCAAACTGGTTGCAG | 121 | 60 |
| *ACOX3* | CGTGGACTTTCTGGATGGCT | CGCTTCTCTCGACTGAGCTT | 159 | 60 |
| *GSTA1* | TCAAGTGGGAGACAGACTTTCG | TTCTGCCGCGTCCATTGAAA | 99 | 60 |
| *GSTM3* | TTACTGGGATATTCGCGGGC | CTTCCCCGCACGTGTATCTT | 95 | 60 |
| *GSTT2* | TGAGACACTGGCGAACACTC | AAGTCCGCCACTGAGATGTG | 114 | 60 |
| *ADH6* | TAGTCTGGAAGCCTGGTGGA | TGGCCACCATCTTTATGCGA | 90 | 60 |
| *PLIN1* | TGGCACTGAGACAACATCAGC | TTGGAGCTCTTCAAGCCTCA | 103 | 60 |
| *ADIPOQ* | AGCCGCTTATGTGTATCGCT | TCATGTACACCGTGATGTGGT | 188 | 60 |
| *FABP3* | ACGCGTTCTCTGTCGTCTTT | AACCGACACCGAGTGACTTC | 124 | 60 |
| *IGHM* | GCTCAGAGAAGGAGATTGACGA | CGTGGCTACGCCCTGATTAT | 173 | 60 |
| *EIF4EBP1* | CTCTTGGGAGAAGCACACCA | GATGGTCGCTTGCATAAGGC | 45 | 60 |
| *FGF10* | GTGCGGAGCTACAATCACCT | GCTGACCTTCCCGTTCTCAA | 96 | 60 |
| *PIK3AP1* | AAGAGCCACTTGTCAGACCG | CTCTTCTGGTTGAGCTGCCA | 181 | 60 |
| *LPAR3* | GCCACTATCCGTAGCTGCTT | CTGGCCACGGTTTGTTTGAG | 111 | 60 |
| *COL1A2* | TGCACCACTTGTGGCTTTTG | GTGTGGATCACACTCACAGGA | 107 | 60 |
| *COL1A1* | TGTCCTAACGCCAAAGTCCC | TCGACTCCGGTGGTTTCTTG | 92 | 60 |
| *FGF9* | TATAGCCGTGGGTCTGGTCA | ACGTAGAACCTCCGTCCAGT | 195 | 60 |
| *TNXB* | ACGAGCAAGGGGATATACCAG | TACCTGCTCTGTCCCGAACT | 98 | 60 |
| *GNGT2* | CATCAAGCCTGCTTCAGGA | AGAGGGTCGTTTCCTGCTTC | 188 | 60 |
| *CREB5* | GGGTTGGAGGCTAGACAGTT | AGTAGCCTTGAACTCCCCAGA | 78 | 60 |
| *PPP2R2C* | TAACGTACGTGTTGTCTGAGGC | TGTGGCGCATTTTTGCTCTC | 191 | 60 |
| *BRCA1* | CCTCAGCCTCTGCACTGAAA | ATGTGAGCAGCAGCTGGATT | 73 | 60 |
| *THBS2* | CCAGATCGACACGGACAACA | GTTGAAGACATCGTCCCCGT | 73 | 60 |

**Supplementary Table 2. Comparative analysis of meat quality characteristics of *Gayal* and yellow cattle.**

| Item | yellow cattle | *Gayal* | SEM | *P*-value |
| --- | --- | --- | --- | --- |
| L _45min_ | 27.28 | 28.92 | 0.50 | 0.10 |
| L _24h_ | 29.24 | 31.02 | 0.96 | 0.37 |
| a _45min_ | 7.10 | 7.87 | 0.19 | 0.03 |
| a _24h_ | 11.01 | 11.98 | 0.23 | 0.03 |
| b _45min_ | 4.66 | 5.09 | 0.21 | 0.32 |
| b _24h_ | 7.65 | 8.55 | 0.46 | 0.35 |
| pH _45min_ | 6.32 | 6.54 | 0.06 | 0.09 |
| pH _24h_ | 5.45 | 5.89 | 0.11 | 0.03 |
| Shear force, N | 47.03 | 46.05 | 0.73 | 0.52 |
| Drip loss, % | 3.39 | 3.52 | 0.11 | 0.56 |
| Cooking loss, % | 42.10 | 41.55 | 0.48 | 0.59 |
| Protein, % | 20.18 | 21.81 | 0.40 | 0.04 |
| Moisture, % | 76.27 | 76.94 | 0.20 | 0.10 |
| intermuscular fat , % | 0.99 | 0.45 | 0.07 | 0.00 |
| Ca, % | 0.05 | 0.05 | 0.00 | 0.92 |
| P, % | 0.15 | 0.14 | 0.01 | 0.23 |
| Ash, % | 1.51 | 1.33 | 0.07 | 0.22 |

**Supplementary table 3. The absolute content of fatty acids in the longissimus dorsi (LD) of *Gayal* and yellow cattle (ng/100g).**

| Item | yellow cattle | *Gayal* | SEM | *P*-value |
| --- | --- | --- | --- | --- |
| C8:0 | 183.77 | 229.66 | 16.37 | 0.17 |
| C10:0 | 127.02 | 152.71 | 10.22 | 0.22 |
| C12:0 | 1357.74 | 1042.38 | 93.05 | 0.09 |
| C15:0 | 351.80 | 320.93 | 30.47 | 0.63 |
| C16:0 | 33820.12 | 27793.98 | 1262.75 | 0.01 |
| C17:0 | 715.82 | 508.26 | 44.66 | 0.01 |
| C18:0 | 37445.12 | 31293.98 | 1870.63 | 0.10 |
| C22:0 | 693.99 | 461.03 | 80.69 | 0.16 |
| C12:1 | 191.65 | 190.36 | 14.60 | 0.97 |
| C14:1 | 425.36 | 361.86 | 29.58 | 0.30 |
| C15:1 | 65.23 | 60.17 | 4.49 | 0.59 |
| C16:1T | 975.28 | 704.8 | 56.10 | 0.01 |
| C16:1 | 6486.87 | 4942.7 | 390.09 | 0.04 |
| C17:1 | 427.03 | 388.36 | 42.65 | 0.67 |
| C18:1(n-9)T | 1659.26 | 1096.21 | 119.54 | 0.01 |
| C18:1(n-7)T | 1113.37 | 951.12 | 148.99 | 0.60 |
| C18:1(n-12) | 608.01 | 516.9 | 77.29 | 0.57 |
| C18:1(n-9) | 16267.02 | 15439.4 | 665.06 | 0.55 |
| C18:1(n-7) | 20049.74 | 19346.25 | 654.47 | 0.61 |
| C19:1(n-9)T | 1250.95 | 1287.35 | 84.43 | 0.84 |
| C20:1 | 157.11 | 218.6 | 15.23 | 0.04 |
| C24:1 | 1450.1 | 1319.88 | 102.27 | 0.54 |
| C18:2(n-6)T | 1113.5 | 1114.05 | 108.62 | 1.00 |
| C18:2(n-6) | 7739.13 | 9879.67 | 599.08 | 0.07 |
| C18:3(n-6) | 544.02 | 720.92 | 66.54 | 0.19 |
| C18:3(n-3) | 2053.61 | 2805.93 | 160.31 | 0.01 |
| C20:2 | 818.32 | 718.13 | 52.29 | 0.36 |
| C20:3(n-3) | 2157.48 | 2835 | 226.21 | 0.14 |
| C22:2(n-6) | 379.57 | 438.61 | 38.25 | 0.46 |
| C22:2 | 692.52 | 1672.24 | 171.37 | 0.00 |
| C22:4 | 399.57 | 718.96 | 55.15 | 0.00 |
| C22:5(n-3) | 1029.61 | 1271.77 | 233.73 | 0.62 |
| C22:5(n-6) | 1445.13 | 1530.63 | 157.90 | 0.80 |
| SFA | 74695.38 | 61802.92 | 2597.60 | 0.01 |
| UFA | 69499.42 | 70529.86 | 1604.76 | 0.76 |
| MUFA | 31077.23 | 27477.71 | 928.26 | 0.04 |
| PUFA | 18372.45 | 23705.90 | 1066.35 | 0.00 |
| n-3 PUFA | 8178.35 | 10915.56 | 565.47 | 0.01 |
| n-6 PUFA | 9776.21 | 12153.25 | 633.14 | 0.06 |

The fatty acid content of which saturated fatty acids (SFA) contained C8:0, C10:0, C12:0, C15:0, C16:0, C17:0, C18:0 and C22:0; monounsaturated fatty acids (MUFA) consisted of C12:1, C14:1, C15:1, C16:1, C16:1, C16:1T, 17:1, C18:1(n-7)T, C18:1(n-12); C18:1(n-7)T, C18:(1n-9T), C18:(1n-7), C18:(1n-9) and C24:1, whereas the unsaturated fatty acids (UFA) were derived by subtracting SFAs from the total fatty acids. Polyunsaturated fatty acids (PUFA) included C18:2(n-6), C18:3(n-3), C18:3(n-6), C20:2, C20:3(n-3), C22:5(n-3), C22:5(n-6) and C22:2(n-6). T: trans.

**Supplementary Table 4. The absolute content of amino acids in the longissimus dorsi (LD) of Gayal and yellow cattle (g/100g).**

| Item | yellow cattle | *Gayal* | SEM | *P*-value |
| --- | --- | --- | --- | --- |
| Aspartic acid | 1.68 | 1.82 | 0.03 | 0.01 |
| Threonine | 0.87 | 0.89 | 0.02 | 0.57 |
| Serine | 0.77 | 0.80 | 0.01 | 0.07 |
| Glutamate | 3.26 | 3.51 | 0.06 | 0.02 |
| Glycine | 0.85 | 0.86 | 0.01 | 0.55 |
| Alanine | 1.05 | 1.15 | 0.02 | 0.00 |
| Cysteine | 0.10 | 0.11 | 0.00 | 0.12 |
| Valine | 1.03 | 1.04 | 0.02 | 0.93 |
| Methionine | 0.21 | 0.22 | 0.01 | 0.32 |
| Isoleucine | 0.91 | 1.09 | 0.03 | 0.00 |
| Leucine | 1.54 | 1.72 | 0.03 | 0.00 |
| Tyrosine | 0.64 | 0.66 | 0.01 | 0.25 |
| Phenylalanine | 0.79 | 0.82 | 0.02 | 0.50 |
| Histidine | 0.83 | 0.97 | 0.03 | 0.01 |
| Lysine | 1.75 | 1.87 | 0.04 | 0.10 |
| Arginine | 1.18 | 1.24 | 0.02 | 0.10 |
| Proline | 0.59 | 0.62 | 0.02 | 0.28 |
| DAAs | 6.90 | 7.57 | 0.12 | 0.00 |
| SAAs | 5.87 | 6.19 | 0.08 | 0.03 |
| EAAs | 7.92 | 8.61 | 0.13 | 0.00 |
| NEAAs | 10.10 | 10.77 | 0.14 | 0.01 |
| TAAs | 18.02 | 19.38 | 0.26 | 0.00 |
| EAAs/NEAAs | 0.78 | 0.80 | 0.01 | 0.15 |
| EAAs/TAAs | 0.44 | 0.44 | 0.00 | 0.15 |

Amino acid analyses in the LD muscle showed that total amino acids (TAAs) consisted of essential amino acids (EAAs) such as Histidine, Isoleucine, Leucine, Lysine, Methionine, Phenylalanine, Threonine, and Valine, as well as non-essential amino acids (NEAAs) including Alanine, Aspartic acid, Arginine, Glutamate, Glycine, Serine, Tyrosine, Proline, and Cysteine. DAAs (Delicious Amino Acids) consist of Aspartic acid, Glutamate, Glycine, Alanine, and Isoleucine; SAAs (Sweet Amino Acids) consist of Glycine, Alanine, Serine, Proline, Lysine and Threonine.

## Supplementary Figures

**
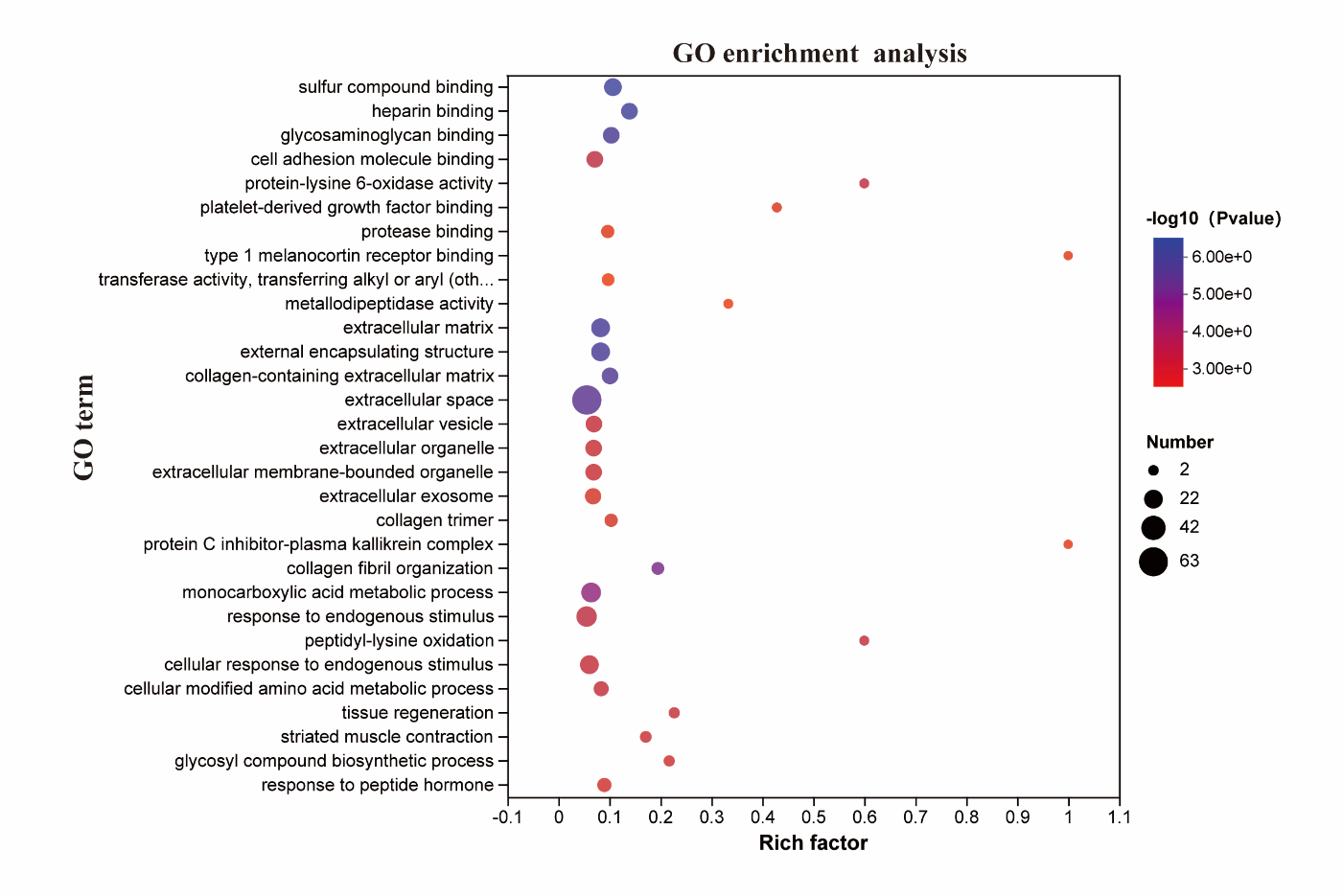
**

**Supplementary Figure 1.** GO analyses of DEGs indicate pathway enrichment.
